# Quantifying spatial gradients in coral reef benthic communities using multivariate dispersion

**DOI:** 10.1098/rsos.241254

**Published:** 2025-04-02

**Authors:** Alice K. Lawrence, Adel Heenan, Gareth J. Williams

**Affiliations:** ^1^ School of Ocean Sciences, Bangor University, Menai Bridge, Anglesey LL59 5AB, UK

**Keywords:** American Samoa, benthic heterogeneity, *Betadisper*, community variability, coral life history, coral species

## Abstract

Tropical coral reefs are dynamic, disturbance-driven ecosystems that are heterogeneous across space and time, partly owing to gradients in cross-scale human impacts and natural environmental factors. Localized management interventions that strive to maintain the long-term persistence and function of coral reefs need to be informed by how and why reef habitats vary. Using the ‘multivariate dispersion’ metric, a statistical approach to measure ecological community variability, we quantified spatial gradients in coral reef benthic communities around Tutuila Island in American Samoa, central South Pacific. Benthic communities with low, medium and high dispersion each had distinct and consistent underlying benthic community characteristics. Low dispersion sites were consistently characterized by high hard coral cover, medium dispersion sites were generally dominated by crustose coralline algae, while high dispersion sites were dominated by turf and fleshy coralline algae. Variability in hard coral and turf algal cover explained 42% of the underlying variation in benthic community dispersion across sites, while site-level gradients in human impacts and environmental factors did not correlate well with variations in benthic community dispersion. The metric should be further tested on temporal data to determine whether it can summarize complex community changes in response to and following acute disturbance.

## 1. Introduction

Tropical coral reefs are dynamic, disturbance-driven ecosystems that display habitat heterogeneity across space and time [1,2]. This heterogeneity is partly driven by gradients in environmental factors like surface wave energy, seawater temperature and differences in nutrient concentrations and primary production [3–6]. These broad-scale environmental gradients cause variation in habitat condition that, in part, dictate which benthic groups can then compete for space at smaller scales on the reef floor [7,8]. Human impacts of varying scale, such as ocean warming, overharvesting of resources, habitat loss and nearshore declines in water quality associated with coastal development, also drive reef ecosystem patterns and processes [9]. These impacts are superimposed over the backdrop of natural environmental factors and together shape coral reef benthic community organization on many contemporary coral reefs [10–12]. Localized management interventions that strive to maintain the long-term persistence and function of coral reefs need to be informed by how and why coral reef habitats vary [13–15]. Attempts to modify reef condition by manipulating manageable human drivers must do so within the natural bounds of the system and what is even achievable given the local environmental context of the reef community [16]. An essential step to achieve this is to effectively quantify and characterize coral reef benthic community heterogeneity across gradients in these various driving forces.

Over the last four decades, multiple stressors on coral reefs have occurred more frequently and at stronger intensities [17], driving global decline in coral cover and habitat complexity [18–20] and changes in ecosystem function [21–23]. Some coral reefs, typically formed by reef-building scleractinian corals, have become dominated by other non-accreting benthic groups (e.g. fleshy macroalgae, soft coral, turf algae and sponges [14,15,24–26]). In some instances, this can lead to ‘biotic homogenization’, whereby multiple specialist species and groups are replaced by fewer, more generalist species and groups to create more spatially homogenous reef communities [12,27,28].

Studies documenting such changes in reef communities have often focused on overall declines in total coral cover, overlooking more taxonomically resolved changes in community structure [12,29–31]. For example, shifts in coral community composition following acute and chronic disturbance can occur because of a disproportionate loss of fragile habitat-forming branching, plating and digitate *Acropora* and *Pocillopora* coral species, compared to the more resilient massive and encrusting coral forms that offer more limited shelter for reef-associated organisms [32–36]. One approach to better understand changes in coral communities beyond changes in total cover is to categorize coral species by their life history strategy. These include the ‘competitively’ dominant, fast-growing species, which are more sensitive to disturbance compared with ‘stress-tolerant’, slower-growing species; the opportunistic ‘weedy’ corals, which quickly recolonize after disturbance; and the ‘generalist’ group of species that display characteristics of the other three strategies [37]. The application of these trait-based groups is one method of characterizing coral reef composition in the face of their natural heterogeneity and in response to acute and chronic disturbance [35,38].

**Figure 1.**
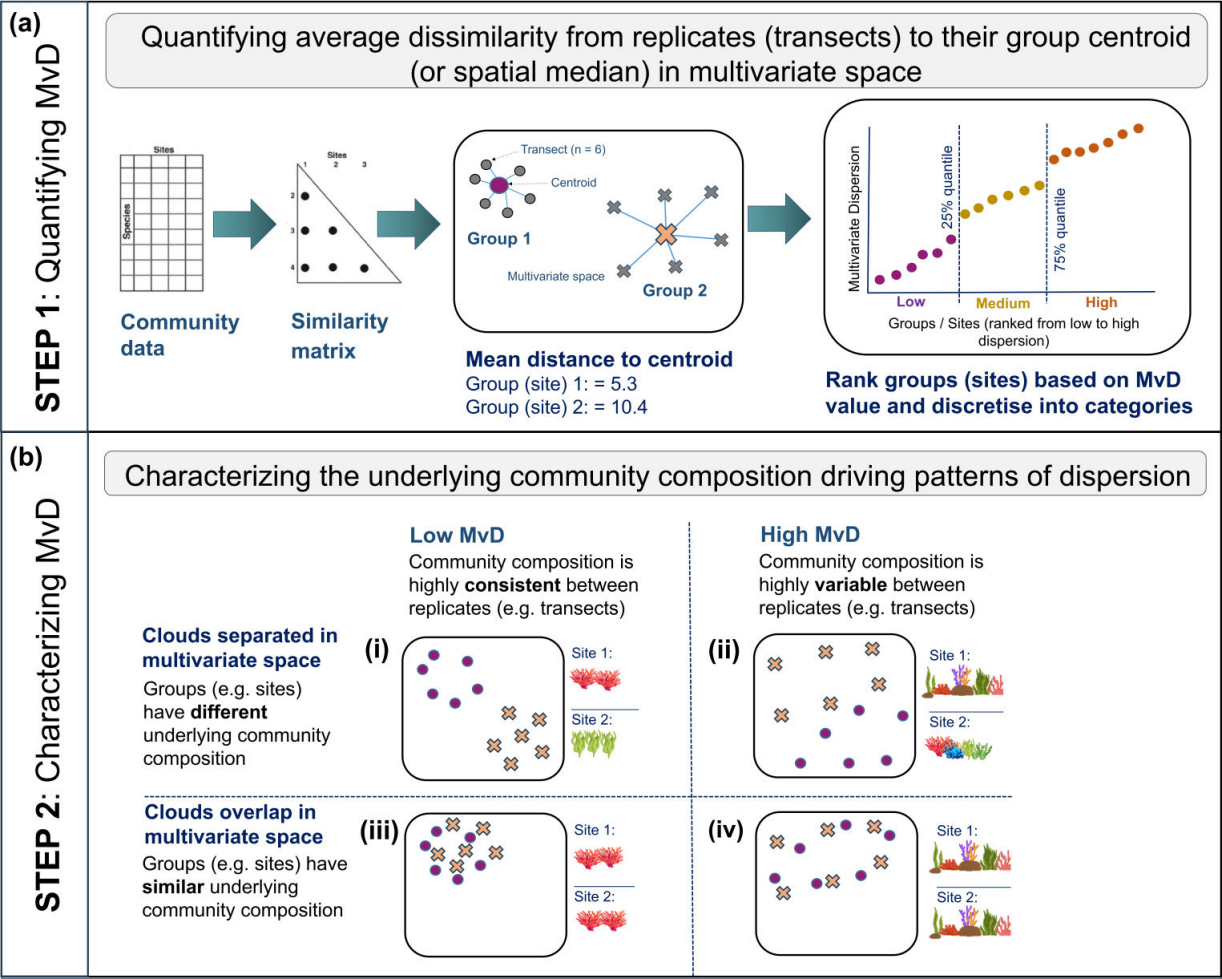
Analytical pipeline used to quantify benthic community multivariate dispersion among observations (in our case, ‘transects’) within each group (in our case, ‘sites’) (*a*: STEP 1) and to characterize the underlying benthic community composition of gradients in dispersion in multivariate space (*b*: STEP 2).

Changes in ecological community composition can also be quantified statistically, and although functional diversity indices are commonly used, there is a need to explore other community-level metrics. Beta diversity, a measure of biodiversity related to species turnover, can be used to estimate the variability in species composition among sampling units for a given area at a given spatial scale [39]. Anderson [40] developed the ‘multivariate dispersion’ metric, as a measure of beta diversity, which quantifies the variability in ecological communities (in multivariate space) among independent sampling units (figure 1).

Low multivariate dispersion indicates that community composition is highly consistent between replicates (e.g. transects) within groups (e.g. sites), whereas high multivariate dispersion is indicative of more heterogeneous communities, with greater replicate-to-replicate variability in community structure. Two groups can, of course, have the same level of multivariate dispersion (e.g. low or high dispersion sites) but for different underlying taxonomic reasons. As such, two groups with similar dispersion levels may overlap or not overlap in multivariate space, indicating that they have similar or different underlying communities respectively (figure 1). Previous works have used changes in multivariate dispersion of ecological communities to indicate environmental stress [41,42], capture the recovery trajectories of coral reefs following warming events [43], quantify depth and latitudinal gradients in temperate reef fish communities [44] and highlight how temperate reef fish communities respond differently to changes in habitat structure at varying spatial scales [45]. Very few studies have applied the multivariate dispersion metric to understand the spatial heterogeneity within and across locations on tropical coral reefs despite the metric having higher sensitivity compared to univariate counterparts in detecting low levels of disturbance [39,43,46]. This synthetic data reduction method has the potential to be used more broadly to understand underlying differences in habitat within the whole community and to characterize the differences that may exist within and between reefs.

Here, we apply and assess the use of the multivariate dispersion metric to characterize coral reef benthic communities. This is an important first step in determining whether the metric is an effective reef resilience monitoring indicator for synthesizing complexities in benthic communities that can inform local management interventions in maintaining the long-term persistence and function of coral reef ecosystems. Using survey data, we quantified the spatial gradients in coral reef benthic community variability across sites around the island of Tutuila in American Samoa, which represent major watersheds along a gradient of reef geomorphologies (steepness and habitat complexity), wave exposures, water quality and human impact. American Samoa has a history of multiple and varied types of disturbance over the past 40 years, including two major coral predator (crown-of-thorns) outbreaks (1976 and 2013), four mass bleaching events (1994, 2002, 2003 and 2017), 10 cyclones, six extreme low tide events and a tsunami in 2009 [47]. The coral reef communities in American Samoa have shown resiliency for rapid recovery and high tolerance to natural and human-induced stressors [47], providing a suitable study area to understand spatial heterogeneity in response to the various driving factors. Specifically, the study aims were to: (i) quantify patterns of benthic community multivariate dispersion across space (sites); (ii) characterize the underlying benthic community composition of gradients in multivariate dispersion (composition of benthic functional groups and coral communities); (iii) test whether the percentage cover of specific benthic groups or metrics of benthic diversity explains patterns of multivariate dispersion across space; and (iv) test whether gradients in human impacts and environmental factors explain patterns of multivariate dispersion across space.

## 2. Material and methods

### 2.1. Study area

**Figure 2.**
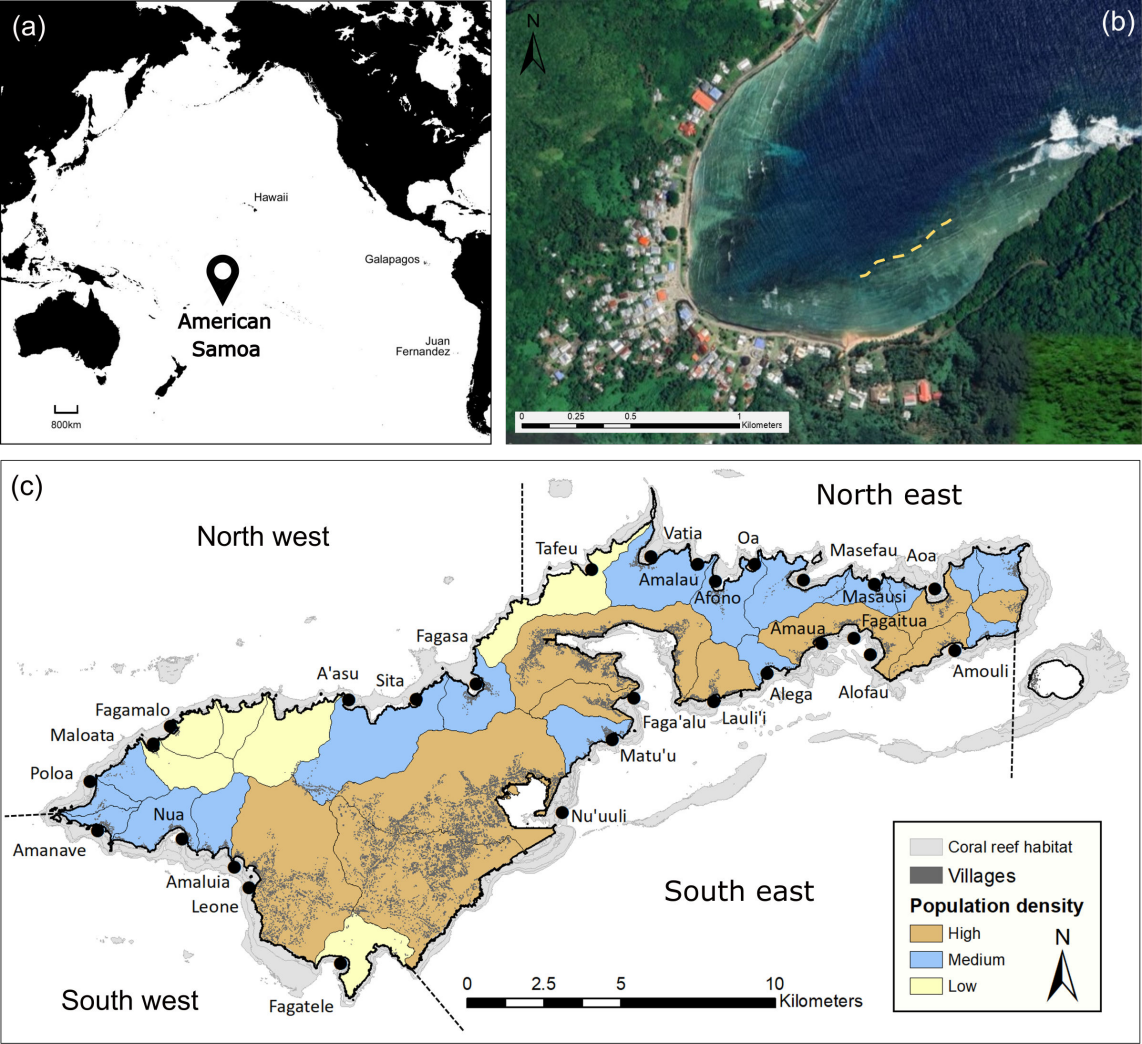
(*a*) Location of American Samoa in the central South Pacific Ocean (black marker). (*b*) Example site surveyed using multiple transects (yellow dotted lines), image source: [48]. (*c*) Survey site locations (displayed with black dots) within the four biogeographical sectors around Tutuila Island (delineated by dotted lines) and categories of major watershed delineations based on human population density (low, medium, high).

Data were collected around the high volcanic island of Tutuila in American Samoa, an unincorporated United States of America Territory located in the central South Pacific Ocean (14.27° S, 170.13° W) (figure 2*a*). Tutuila Island has a human population of approximately 56 000, a total land area of approximately 200 km^2^ and a forereef habitat area (the outer reef slope facing the open ocean) of approximately 49 km^2^ [49]. Surveys were conducted over a three week period in November 2016, as part of an inter-agency watershed monitoring project, which aimed to integrate existing coral reef surveys and water quality sampling conducted by local government agencies [50]. As part of the project, 28 sites were chosen using ArcMap 10.4 to represent major watershed delineations around Tutuila (figure 2*c*). To ensure comparability, survey sites were located in bays on the forereef habitat at 10 m depth and approximately 250 m out from any major stream mouth (figure 2*b*). Human population density per major watershed was calculated from the 2010 census of American Samoa using the population counts for places (villages), and each site was categorized into low (≤ 25th percentile), medium (≥ 25th and ≤ 75th percentile) or high (≥ 75th percentile) human population [51]. Sites were categorized into four geographical sectors (northwest, northeast, southwest, southeast), based on biogeographic habitat delineations used by the National Oceanic and Atmospheric Administration’s (NOAA’s) Pacific Reef Assessment and Monitoring Program ([52]; figure 2*c*).

### 2.2. Benthic community digital surveys and post-processing

At each site, surveys were conducted by divers on SCUBA by laying two 100 m transect tapes consecutively along the 10 m depth contour parallel to shore in the direction of the open ocean (figure 2*b*). Benthic community surveys were then conducted along six 25 m sections of this combined 200 m linear distance with 5 m breaks in between each of them: 0−25, 30−55, 60−85, 90−115, 120−145 and 150−175 m. Along each 25 m section, digital images of the benthos were taken approximately 1 m above the sea floor at 1 m intervals using an Olympus Tough TG-4 camera (*n* = 26 images taken per transect, *n* = 156 images per site).

For each image, five randomly allocated points were overlaid (*n* = 125 data points per transect, 750 data points per site; [53]) using Coral Point Count with Excel extensions [54] and the substrate under each point identified as belonging to one of the following 10 major categories: hard coral (to genus level and growth forms within genera such as *Acropora* ‘tables’, ‘staghorn’ or ‘arborescent’); crustose coralline algae (CCA; multiple genera); branching coralline algae; non-calcified macroalgae (greater than 2 cm, to genus level if abundant); *Halimeda* spp. (a common genus of calcifying macroalgae across the Pacific); turf algae (a mixed community of filamentous algae and cyanobacteria less than 2 cm tall, including the ‘epilithic algal matrix’); fleshy coralline algae (e.g. shedding-calcareous algae known to overgrow corals like *Peyssonnelia* spp. [55]); other invertebrates (including sponges, and soft coral to genus level if abundant); sand and rubble (electronic supplementary material, table S1). This categorization resulted in 61 minor categories, 41 of which were coral genera and common coral species within the hard coral major category (electronic supplementary material, table S2). The benthic substrate ratio (BSR) can be used as a metric of reef condition [56] by calculating the ratio of heavily calcified organisms (hard corals, CCA, branching coralline algae and *Halimeda* spp.) to less- or non-calcifying (turf algae, non-calcified macroalgae and fleshy coralline algae) benthic variables for each survey site. Coral genera and common coral species were classified into four different life-history strategy categories: competitive, opportunistic weedy, stress-tolerant and generalist, which are primarily separated by colony morphology, growth rate and reproductive mode (*sensu* [37]; electronic supplementary material, table S2). Key coral genera were also classified into rapid- and slow-growing categories [35], based on the growth forms ‘bushy and tabular’, and ‘massive and columnar’ (electronic supplementary material, table S2).

### 2.3. Quantifying human impacts and environmental factors

Human impacts and environmental factors collated for each survey site included surface wave energy, dissolved inorganic nitrogen, human population density per major watershed, the proportion of disturbed land in each major watershed, reef steepness and habitat complexity. Surface wave energy, a key driver of benthic community structure on coral reefs [4,57], was calculated using a wave exposure proxy developed for Tutuila by NOAA [58], which is an estimate of the mean maximum daily wave power (kW m^−1^) over a 10-year period (2002−2012) at 1 km resolution using the NOAA WaveWatch III global wave model (http://pacioos.org/metadata/as_noaa_all_wave_avg.html). Dissolved inorganic nitrogen was used as a proxy of ‘water quality’ owing to it often being the most abundant and bioavailable form of nitrogen and relatively straightforward and economical to analyse [59]. Dissolved inorganic nitrogen concentrations (in mg l^−1^) were measured using a SEAL Analytical AA3 HR nutrient analyzer [50]. Mean, standard deviation and maximum dissolved inorganic nitrogen were calculated for each survey site using data from samples collected at 26 streams, which were located within major watersheds associated with each survey site. The samples were collected at the same time each month over a 12-month period between September 2016 and September 2017 with a few exceptions. Two of the survey sites were only sampled twice, and another two sites were not sampled at all owing to the inaccessibility of the stream from land. As each sample represents a snapshot in time, we calculated the 12 month mean, standard deviation and maximum value for each site to account for any seasonal variations in rainfall and storm events. To try and capture local human impacts to the nearshore reefs, we quantified two proxies: human population density and nearby land use. Human population density per major watershed was calculated from the 2010 census of American Samoa using the population counts for places (villages) (https://www.census.gov/population/www/cen2010/island_area/as.html). The proportion of disturbed land to undisturbed land in each major watershed’s area was estimated in ArcGIS 10.4 using the American Samoa vegetation layer derived from QuickBird satellite imagery [60]. The total area of disturbed land was calculated using four categories: quarry/landfill (areas recently bulldozed for quarrying activities or used for solid waste disposal), secondary scrub (an intermediate type of vegetation that occurs when cultivated land is abandoned and allowed to revert to natural forest), urban built-up (impervious urban surfaces such as houses and paved roads) and urban cultivated area (all vegetated areas within a general urban boundary). To quantify site-level habitat complexity, four digital images were taken of the reefscape at the start of each transect at each site, by facing each major cardinal direction (N, E, S, W). Each image was visually and manually scored from 0 to 5, where 0 = no vertical relief; 1 = low and sparse relief; 2 = low but widespread relief; 3 = moderately complex; 4 = very complex with numerous fissures and caves; 5 = exceptionally complex with numerous caves and overhangs [61]. Site-level reef steepness was also estimated using the same images, by assigning a value from 1 to 5, where 1 = flat, 2 = gradual slope, 3 = 45° slope, 4 = 65° slope and 5 = vertical wall. These transect-level values of habitat complexity and steepness were then used to calculate site-level averages.

### 2.4. Statistical analyses

To quantify variability in community composition (multivariate dispersion) across the six benthic transects at each site, we used the ‘*betadisper’* function in the *vegan* package [62] for R (https://www.r-project.org). The ‘*betadisper*’ function runs a distance-based test for the analysis of multivariate homogeneity of group dispersions (variances; [40,46]) and calculates the distance of each observation (in this case ‘transect’, *n* = 6) to its group centroid (in this case ‘site’, *n* = 28). We used distance to spatial median as our distance measure (the point in the multivariate cloud that minimizes the sum of the distances from each replicate observation to that point) as it is less affected by outliers [63]. Calculations of multivariate dispersion were run on a Euclidean similarity matrix for the mean percentage cover of the 10 major benthic variables. No transformations were applied to the data to preserve the raw dispersion among transects within each site [40]. Patterns of multivariate dispersion were visualized using non-metric multi-dimensional scaling (nMDS) using the *metaMDS* function in the *vegan* package [62], again using Euclidean similarity matrices for the major benthic variables. Sites were ranked based on their distance to median (dispersion) values, which were defined as low (≤ 25th percentile), medium (≥ 25th and ≤ 75th percentile) or high (≥ 75th percentile) dispersion categories.

To investigate which of the benthic characteristics and human impacts and environmental factors (predictor variables) best explained variation in multivariate dispersion at the major benthic category taxonomic resolution (response variable), we used distance-based linear modelling (DISTLM [64,65]). In addition to the benthic variables and human impacts and environmental factors, we calculated a suite of diversity indices on both the mean percentage cover of the major benthic variables and the coral genera data using the DIVERSE function in PRIMER v. 7.0.23 [66]. The indices calculated for each site were Margalef’s species richness (*d*); the Shannon–Wiener index (*H*’), which places more emphasis on rare or less abundant variables; Simpson’s index (*λ*), which places more emphasis on the more dominant variables [63] and Pielou’s evenness (*J*), which measures how uniformly spread the total abundance of each variable is within each observation [66]. Prior to model fitting, we tested whether any of the predictor variables were significantly correlated with each other using the ‘ggcorrplot’ package in R [67], testing the null hypothesis that each pairwise comparison was not correlated (electronic supplementary material, figures S1 and S2). The following predictors significantly correlated: Shannon’s diversity index of the major and minor benthic substrate groups correlated with the Simpson’s diversity index (*r* = 1), we retained the Shannon diversity index as it emphasizes less abundant species instead of dominant species; Pielou’s evenness of benthic groups and Simpson’s diversity index of benthic groups (*r* = 0.9), we retained Pielou’s evenness of benthic groups; sand and rubble (*r* = 0.9), rubble was retained owing to the relative importance of rubble with regard to benthic invertebrate diversity [68]; and mean correlated with maximum dissolved inorganic nitrogen (*r* = 0.9). We retained maximum dissolved inorganic nitrogen, given that maximum exposure to nutrient stress is likely to be more important than mean exposure. The final suite of benthic variables and human impacts and environmental factors included in the models are listed in table 1.

**Table 1.** Predictor variables, biotic (*a*) and human impacts and environmental (*b*), used to try and explain variation in coral reef benthic community multivariate dispersion among sites using distance-based linear modelling (DISTLM). (Units and spatial/temporal resolution are shown for each variable and the data sources for the human impacts and environmental factors.)

(a) Biotic variables	Unit
Branching coralline algae	%
Benthic Substrate Ratio (BSR)	ratio
Coral	%
Crustose Coralline Algae (CCA)	%
Evenness - J (Major Benthic)	
Evenness - J (Coral Genera)	
Fleshy coralline algae	%
*Halimeda* spp. (calcified algae)	%
Macroalgae (non-calcified)	%
Other invertebrates	%
Rubble	%
Shannon diversity - H’ (Benthic)	
Shannon diversity - H’ (Coral)	
Turf algae	%

(*b*) Human impacts and environmental factors	Units	Resolution	Time period	Source
Dissolved inorganic nitrate – mean	mg l^−1^	monthly	09/16 to 09/17	R2R project [46]
Dissolved inorganic nitrate –standard deviation	mg l^−1^	monthly	09/16 to 09/17	R2R project [46]
Dissolved inorganic nitrate – maximum	mg l^−1^	monthly	09/16 to 09/17	R2R project [46]
Disturbed land	%		2010	QuickBird imagery [56]
Habitat complexity	index		2017	R2R project [46]
Human population density	people km^−2^	village	2010	2010 census (https://www.census.gov/population/www/cen2010/island_area/as.html)
Reef steepness	index		2017	R2R project [46]
Wave energy (mean)	kW m^−1^	1 km	2002−2012	NOAA Wave Watch III (http://pacioos.org/metadata/as_noaa_all_wave_avg.html)

Models were first built using the benthic characteristics as the predictor variables, and then the model-fitting process was repeated using the human impacts and environmental factors as predictors. In each case, the DISTLM models were built from a Euclidean similarity matrix of the site dispersion values. All possible candidate models (i.e. unique combinations of the predictor variables) were computed using the ‘best’ model selection procedure [63] and ranked using Akaike’s information criterion [69] with a second-order bias correction applied (AICc; [70]) to account for the relatively small sample size relative to the number of predictor variables. All models within 15% AICc of the top model are reported, and the marginal relationships between each predictor and benthic dispersion were plotted to identify the overall directionality of the relationships and Pearson’s correlations calculated. All DISTLM analyses were completed using the PERMANOVA+ add on [63] for PRIMER v. 7.0.23 [71]. Source code available at https://github.com/alicelawrence2021/dispersion.git.

## 3. Results

### 3.1. Intra-island gradients in benthic cover

**Figure 3.**
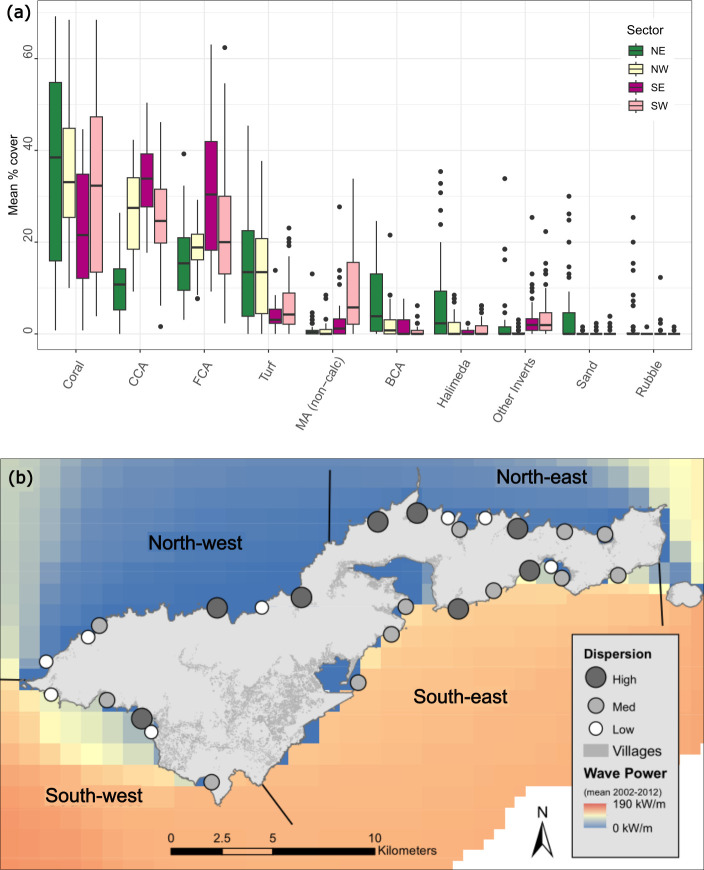
(*a*) Median percentage cover of benthic groups within the four biogeographical sectors northeast (*n* = 8 sites), northwest (*n* = 6 sites), southeast (*n* = 9 sites), southwest (*n* = 5 sites). CCA, crustose coralline algae; FCA, fleshy coralline algae; MA (non-calc), macroalgae (non-calcified); BCA, branching coralline algae; Halimeda, *Halimeda* spp.; Other Inverts, other invertebrates. Black dots represent outliers, and boxes show the interquartile range and their middle lines represent median values. (*b*) Location of the 28 survey sites around Tutuila Island and their associated multivariate dispersion (distance to median) category (low, medium, high), mean maximum daily wave power (kW m^−1^) from 2002 to 2012, location of villages and biogeographic sector delineations.

There were clear intra-island gradients in benthic group cover within the four biogeographical sectors (northeast, northwest, southeast, southwest; (figure 3*a*)). Mean (± s.e.) hard coral cover peaked in the northeast (35.6% ± 7.4%) and was lowest in the southeast (22.4% ± 4.9%) (figure 3*a*). Sites in the northeast also had the highest mean cover of branching coralline algae (7.2% ± 2.4%), *Halimeda* spp. (6.6% ± 3.4%), rubble (2.2% ± 1.6%), sand (4.0% ± 2.4%) and turf algae (14.9% ± 3.9%). The mean cover of turf algae was also high in the northwestern sites (14.1% ± 3.8%) and lowest in the southeast (3.8% ± 0.5%). Sites in the southeast had the highest mean cover of CCA and fleshy coralline algae (33.5% ± 2.0% and 30.8% ± 5.5% respectively). The highest mean cover of non-calcifying macroalgae was at southwestern sites (9.2% ± 2.8%) and lowest in the northwest (0.9% ± 0.5%). The benthic substrate ratio did not identify any island-wide trends in calcifying to non-calcifying organisms by sector, with the highest ratio in the southwest (2.4% ± 0.8%) and lowest in the southeast (1.9% ± 0.5%; electronic supplementary material, table S3).

### 3.2. Gradients in benthic community multivariate dispersion

**Figure 4.**
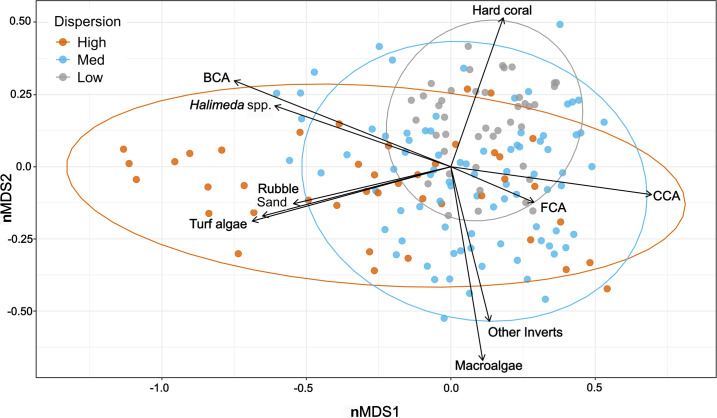
Variation in benthic group cover among multivariate dispersion categories (low, medium, high). Relative similarity in site-level (*n* = 6 transects per site) multivariate dispersion of benthic communities across 28 sites around Tutuila Island, American Samoa. nMDS was constructed from all six transect replicates at each survey site, using Euclidean dissimilarities of non-transformed mean percentage cover estimates of all major benthic categories (stress value: 0.18). The correlation between each benthic variable and the first two ordination axes is overlaid as a bi-plot, with the length of each vector line proportional to the strength of the correlation. CCA, crustose coralline algae; FCA, fleshy coralline algae; BCA, branching coralline algae; Other Inverts, other invertebrates.

**Figure 5.**
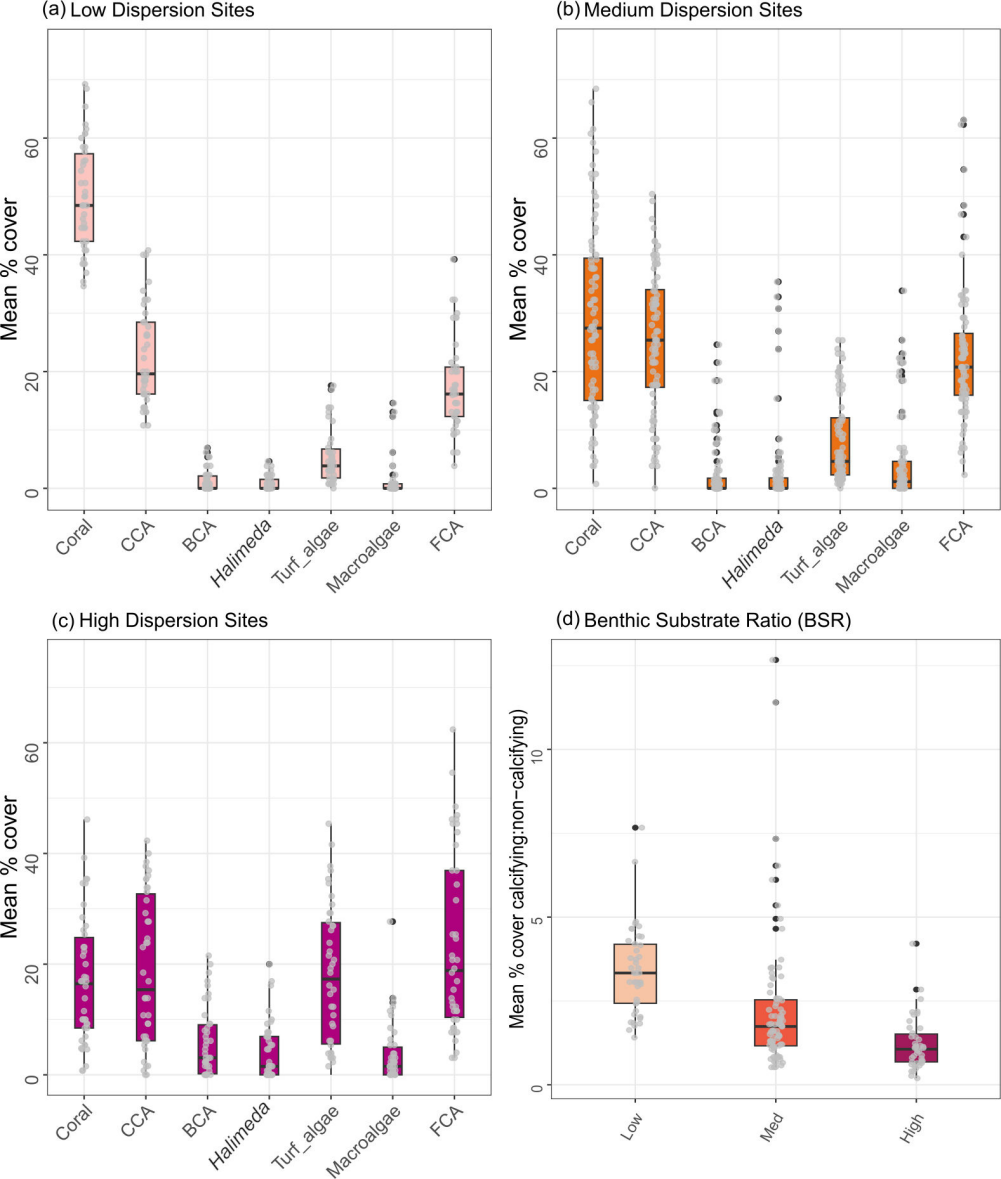
Variation in mean percentage cover of the main benthic substrate categories within each multivariate dispersion category: (*a*) low, (*b*) medium (med), and (*c*) high. The ratio of mean percentage cover of heavily calcified organisms to less- or non-calcifying within each multivariate dispersion category is shown in plot (*d*) benthic substrate ratio (BSR). Boxplots are overlaid with transect replicate data for each survey site, black dots represent outliers and boxes show the interquartile range and their middle lines represent median values. CORAL, hard coral; CCA, crustose coralline algae; BCA, branching coralline algae; HALI, Halimeda spp.; TURF, turf algae; MA, macroalgae; FCA, fleshy coralline algae.

At the site level, low-dispersion sites were characterized as having a higher percentage cover of hard coral (49.9% ± 1.4%), compared to medium (20.0% ± 1.8%) or high (17.1 ± 1.8 %) dispersion sites (figures 4 and 5*a*). The medium- to high-dispersion sites had a mixture of benthic substrate groups, including turf algae, branching coralline algae, macroalgae, sand and rubble (figure 4). The cover of turf algae, *Halimeda* spp., and branching coralline algae was highest at high dispersion sites (17.8% ± 2.0%, 4.1% ± 0.8% and 5.9% ± 1.0% respectively) as compared to low-dispersion sites (5.4% ± 0.7%, 0.9% ± 0.2% and 1.3% ± 0.3% respectively). CCA cover was highest at medium dispersion sites (25.3% ± 1.3%), and lowest at high dispersion sites (18.6% ± 2.1%) (figure 5*b*). CCA cover exceeded hard coral cover (by between 10% and 28%) at 7 of the 28 survey sites, six of which had medium dispersion (see the electronic supplementary material, figure S3 for site-level graphs). Overall, the benthic substrate ratio decreased with increasing dispersion (figure 5*d*), suggesting that low-dispersion sites had a higher proportion of calcifying, reef-building organisms. However, there was no consistent pattern in benthic community multivariate dispersion within and between the four island sectors (electronic supplementary material, table S3).

### 3.3. Gradients in hard coral community multivariate dispersion

**Figure 6.**
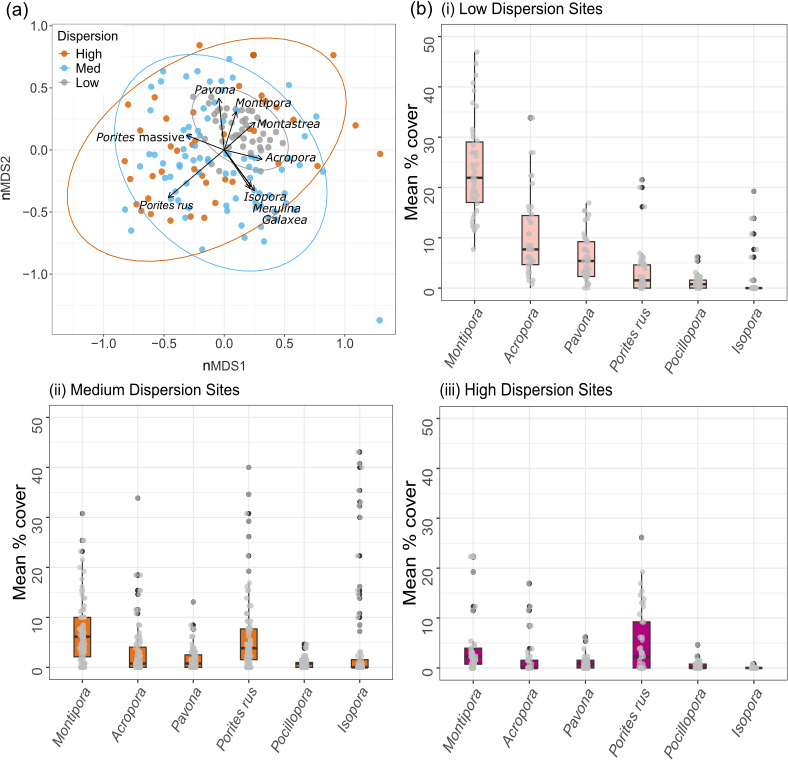
Variation in percentage cover of corals that best discriminated amongst the different multivariate dispersion categories (low, medium, high). (*a*) Relative similarity in site-level (*n* = 6 transects per site) multivariate dispersion of benthic communities across 28 sites around Tutuila Island, American Samoa. nMDS plot based on all six transect replicates at each survey site, using Bray–Curtis dissimilarities of non-transformed mean percentage cover estimates of all coral genera categories (stress value: 0.28). The correlation between each benthic variable and the first two ordination axes are overlaid as a bi-plot, with the length of each vector line proportional to the strength of the correlation. (*b*) Median percentage cover of six coral genera within each benthic dispersion category (i) low, (ii) medium; and (iii) high. Boxplots are overlaid with transect replicate data for each survey site; black dots represent outliers and boxes show the interquartile range and their middle lines represent median values.

The corals that best discriminated among the high-medium-low dispersion categories were *Montipora, Pavona, Acropora* branching and corymbose growth forms, and *Porites rus* (figure 6*a*; see the electronic supplementary material, figure S4 for site-level graphs). Low-dispersion sites were dominated by the encrusting coral *Montipora grisea* (figure 6*b*), where mean cover (23.8% ± 1.5%) was 16.5% higher than at medium-dispersion sites (7.3% ± 0.7%) and 19% higher than at high-dispersion sites (4.7% ± 1.1%). The cover of *Pavona* and all *Acropora* growth forms were also highest at low dispersion sites (6.1% ± 0.7% and 8.1% ± 0.7% respectively; figure 6*b*). *Pocillopora* corals were present in similar abundances at both low- and medium-dispersion sites (1.2% ± 0.2% and 0.8% ±0.1% respectively), and the cover of *Isopora* and *Por. rus* corals was highest at medium-dispersion sites (4.7% ± 1.2% and 6.4% ± 0.9% respectively; figure 6*b*). The mean percentage cover of coral at high dispersion sites was relatively low (18.5% ± 15.0%), with the communities dominated by *Montipora, Pavona* and *Por. rus* (4.7% ± 1.1%, 1.0% ± 0.2% and 5.2% ±1.0% respectively; figure 6*b*).

**Figure 7.**
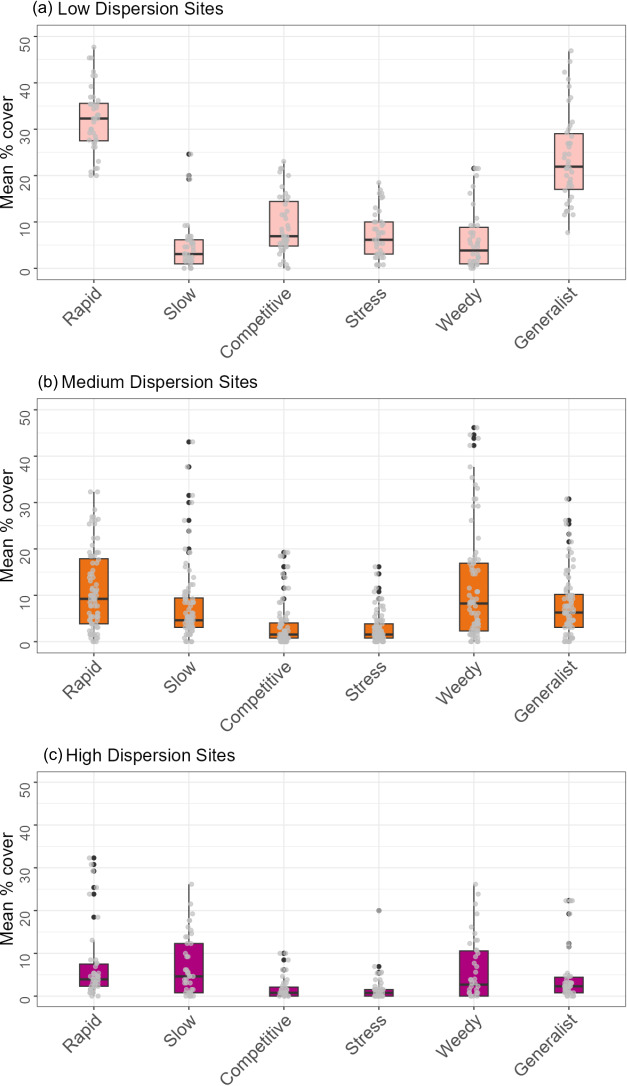
Variation in cover of corals with different life-history strategies among multivariate dispersion categories (low, medium, high). Summary boxplots showing median percentage cover of life history categories within each benthic dispersion category (*a*) low, (*b*) medium, and (*c*) high. Boxplots are overlaid with transect replicate data for each survey site; black dots represent outliers and boxes show the interquartile range, and their middle lines represent median values.

There were also clear patterns in the cover of hard corals with different life-history strategies across dispersion categories (figure 7). The cover of rapid-growing corals was higher at low-dispersion sites (33.0% ± 5.0%) compared to high-dispersion sites (4.2% ± 3.5%; figure 7). The cover of slow-growing corals was higher at medium- and high-dispersion sites (4.8% ± 4.5% and 4.8% ± 6.5% respectively) compared with low-dispersion sites (3.5% ± 5.2%). The mean cover of generalist, competitive and stress-tolerant corals was highest at low dispersion sites (22.0% ± 7.5%, 7.0% ± 12.2% and 6.0% ± 4.3% respectively), and all three groups decreased in cover with increasing dispersion (figure 7). Medium dispersion sites had the highest cover of opportunistic weedy coral species (such as *Por. rus* and *Pocillopora* corals; 8.0% ± 8.2%), followed by high (4.0% ± 0.4%) and then low-dispersion sites (2.5% ± 4.2%; figure 7).

### 3.4. Ecological drivers of multivariate dispersion among sites

Variations in hard coral and turf algae cover (top-performing model) explained 41.5% of the underlying variation in benthic community multivariate dispersion across the 28 sites (table 2).

**Table 2.** Distance-based linear modelling (DistLM) results testing for relationships between benthic community multivariate dispersion across sites (*n* = 28) and underlying benthic community characteristics. (All possible candidate models were run (unique combinations of the predictor variables), and models were ranked using Akaike information criterion with a second-order-bias-correction applied (AICc). All models within 15% AICc of the top-performing model are reported. Proportion (prop. %), overall variation in multivariate dispersion explained by the candidate model (individual contribution of each predictor to the overall model performance is shown in parentheses for each predictor within each candidate model); RSS, residual sum of squares.)

AICc	prop. (%)	RSS	candidate model
53.40	41.47	146.81	turf algae (30.3%), coral (11.1%)
54.11	45.54	136.59	Shannon diversity (benthic) (23.6%), Shannon diversity (coral) (13.9%), macroalgae (8.0%)
54.34	39.46	151.86	turf algae (30.3%), fleshy coralline algae (9.1%)
54.37	45.03	137.76	turf algae (30.3%), coral (11.1%), evenness (coral genera) (0.5%)
54.54	44.71	138.69	turf algae (30.3%), fleshy coralline algae (9.1%), other invertebrates (1.6%)

**Figure 8.**
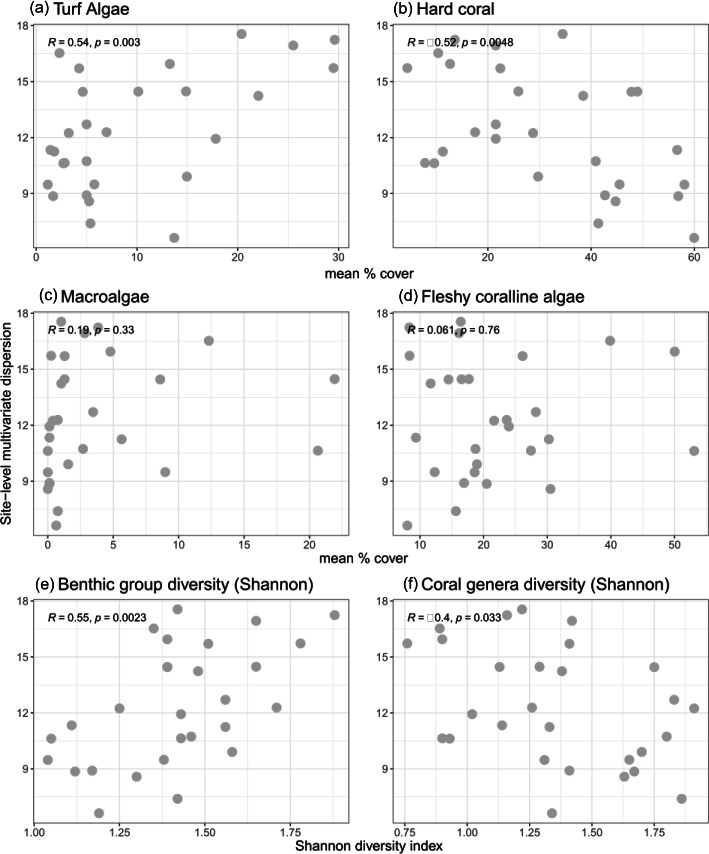
Correlations between benthic community multivariate dispersion (site-level, *n* = 28, mean distance to median) and underlying benthic community characteristics, selected from DISTLM model results. *R, Pearson correlation coefficient; *p*, *p*‐value.

Benthic community multivariate dispersion was negatively correlated with hard coral cover and positively correlated with turf algae cover (figure 8). Variations in coral genera diversity, benthic substrate group diversity and macroalgae explained 45.5% of the underlying variation, and the cover of turf algae and fleshy coralline algae explained 39.5% of the variation in benthic community dispersion. Benthic dispersion positively correlated with mean cover of turf algae and benthic substrate group diversity (figure 8). Conversely, benthic dispersion was negatively correlated with hard coral cover and coral genera diversity (figure 8).

### 3.5. Correlations between benthic community multivariate dispersion and human impacts and environmental factors

Overall, the variation in site-level benthic community multivariate dispersion was not well explained by the human impacts and environmental factors we quantified. Variations in benthic habitat complexity, reef steepness and population density (the top three performing models) explained only 10.2, 7.4 and 7.3% of the overall variability in benthic community multivariate dispersion respectively (table 3). The combination of benthic habitat complexity with reef steepness explained 14.7% of the variation in multivariate dispersion across sites. Similarly, the combination of benthic habitat complexity with population density, and with mean wave power explained 13.6 and 11.9% of the variation in multivariate dispersion respectively. Benthic community multivariate dispersion was negatively correlated with habitat complexity; there were weak positive correlations between benthic dispersion and reef steepness and with human population density (electronic supplementary material, figure S5). Dissolved inorganic nitrate and disturbed land only explained 0.0003 and 1.15% of the overall variation in multivariate dispersion respectively.

**Table 3.** Distance-based linear modelling (DistLM) results testing for relationships between benthic community multivariate dispersion across sites (*n* = 28) and human impacts and environmental factors. (All possible candidate models were run (unique combinations of the predictor variables), and models were ranked using Akaike information criterion with a second-order-bias-correction applied (AICc). All models within 15% AICc of the top-performing model are reported. Proportion (prop. %), overall variation in multivariate dispersion explained by the candidate model (individual contribution of each predictor to the overall model performance is shown in parentheses for each predictor within each candidate model); RSS, residual sum of squares.)

AICc	prop. (%)	RSS	candidate model
62.854	10.213	225.2	habitat complexity (10.2%)
63.703	7.4479	232.14	reef steepness (7.4%)
63.748	7.2999	232.51	human population density (7.3%)
63.929	17.631	213.87	habitat complexity (10.2%), reef steepness (7.4%)
64.294	17.511	216.68	habitat complexity (10.2%), human population density (7.3%)
64.854	11.866	221.06	habitat complexity (10.2%), wave energy (mean) (0.08%)

## 4. Discussion

Using multivariate dispersion, we quantified spatial gradients in coral reef benthic community variability around the circumference of American Samoa in the central South Pacific and investigated whether different dispersion levels (low, medium, high) had commonalities in their underlying benthic community characteristics (figure 1). We found that variability in hard coral and turf algae cover explained most of the underlying variation in benthic community dispersion across sites. Low-dispersion sites were consistently characterized by high coral cover, dominated by encrusting corals and a diverse assemblage of rapid-growing, branching and corymbose coral genera in low abundances. Medium dispersion sites were generally dominated by CCA and coral genera with opportunistic life-history strategies while high dispersion sites were dominated by turf algae and fleshy coralline algae. There was higher cover of calcifying organisms at low dispersion sites, which decreased as dispersion increased. Variations in benthic community dispersion were not well explained by gradients in the human impacts and environmental factors modelled here (< 15% total variation explained), suggesting that smaller scale biological processes may be more important in driving these patterns.

Low-dispersion sites around our study island were consistently dominated by high coral cover rather than macroalgae, turf algae or soft corals that often characterize more homogenous benthic communities on coral reefs subjected to chronic and acute disturbance [24,72]. Sites with low dispersion were dominated by the encrusting hard coral *M. grisea*, which has rapid-growing, stress-tolerant and competitive life-history traits [37]. Low-dispersion sites were also characterized by a high diversity of other predominantly rapid-growing coral genera, all co-occurring in relatively low abundances, including tabulate *Acropora* corals and other branching corals such as *Pocillopora* and *Porites cylindrica*. Although rapid-growing corals with branching and corymbose growth forms tend to be susceptible to thermal stress [32,34,73], and are selectively fed on by coral predators such as crown-of-thorns starfish [74], they are competitively dominant corals that can propagate through fragmentation following acute physical disturbance from storms and persistent high wave energy [4,33,75]. Low-dispersion sites also had the highest cover of *Pavona* corals, which have slow-growing and stress-tolerant life-history strategies [37]. It is unclear why low-dispersion sites were characterized by a diverse mix of rapid-growing and stress-tolerant coral genera. One hypothesis is that low-dispersion sites may be indicative of locations that have experienced both acute and chronic disturbances and may represent areas with environmental conditions that naturally create spatially heterogeneous habitats and diverse and resilient coral communities. Further temporal studies are required to better understand the interactions between different disturbance events and community dynamics at these low-dispersion sites.

Benthic community dispersion increased as the cover of non-reef-building organisms, such as turf algae, fleshy coralline algae and non-calcifying macroalgae, increased, and as overall habitat structural complexity decreased. Unlike low-dispersion sites that consistently had the same underlying benthic community characteristics (figure 1*b*(iii)), the benthic communities creating either medium or high dispersion were highly variable (figure 1*b*(ii)). Medium-dispersion sites had the highest mean cover of CCA, which rapidly colonize bare substrate following disturbance [47], stabilizing the reef [76,77] and providing substrate for coral settlement and growth [47,78]. Medium dispersion sites also had the highest cover of opportunistic weedy corals, including *Por. rus*, which have brooding reproduction and high population turnover [79] that rapidly colonize newly available space following acute disturbance [80]. Long-term monitoring surveys in American Samoa have shown a general decline in the cover of *Acropora* corals and a widespread increase in the cover of *Por. rus* because of disturbances (C. Birkeland 2024, personal communication), which could indicate that medium dispersion sites at this location are characteristic of benthic communities in recovery following acute disturbance. With increased frequency and magnitude of acute disturbances, systems may tend to shift towards earlier successional states [81], which are characterized by simple, low-ecosystem complexity composed of early colonizers that are quick to respond and react to the change in environmental conditions [13]. The high cover of turf algae and fleshy coralline algae at high dispersion sites suggests these sites are dominated by organisms that have colonized newly available space following acute disturbance [82,83], and environmental conditions may not be as favourable as medium dispersion sites.

Over the last decade, fleshy coralline algae, or peyssonnelid algal crusts, have become spatially dominant across shallow reefs in the Caribbean [84], probably owing to their ability to overgrow hard corals [84] and inhibit coral settlement [85]. In the absence of sufficient herbivorous fishes to maintain cropped algal turfs, sediment can accumulate, which inhibits coral settlement and recruitment and may provide suitable conditions for fleshy macroalgae to dominate the benthic community [86,87]. High-dispersion sites had the lowest cover of hard coral, and of the corals present, the highest cover of the large, slow-growing, stress-tolerant *Porites* massive corals. Massive and encrusting coral growth forms such as massive *Porites* and faviids are less susceptible to acute stressors such as coral bleaching [34,73], and can dominate the reef when faster-growing *Acropora* species are unable to recover owing to repeated disturbance [88]. One hypothesis is that high-dispersion sites are in areas with unfavourable environmental conditions and ongoing chronic stress (e.g. human or abiotic), which could contribute to a slower-than-expected recovery (two-phase recovery) following acute disturbances [89]. Massive and encrusting coral growth forms can be more tolerant to variable and chronic stressors [90–92], although there are exceptions to this generalization [93].

Across our study sites, underlying variation in benthic community dispersion was only weakly explained by concurrent gradients in three human impacts and environmental factors: benthic habitat complexity, reef steepness and human population density. As habitat structural complexity increased, benthic dispersion values decreased. Habitat complexity is driven by the underlying benthic community, and at sites with lower dispersion, we saw an increase in coral types that generate higher structural complexity (e.g. tabulate, branching and corymbose corals). There was a weak positive correlation between human population density and benthic community dispersion, where sites close to the highest human population densities around Tutuila had the highest dispersion, relatively low coral cover and habitat structural complexity and high cover of turf and macroalgae. These drivers only explained a small proportion of the variation in multivariate dispersion, yet many studies have found links between local human impacts and a reduction in reef resilience. For example, a decrease in habitat complexity and an increase in fleshy algae cover from overfishing [14,86], nutrient and wastewater pollution [15,94] and coastal development [95]. Additionally, we did not find any associations between variation in benthic community dispersion and surface wave energy, dissolved inorganic nitrogen (water quality proxy) or the proportion of disturbed land in the watershed. Potential explanations are scale mismatches between the spatial resolution of our human impacts and environmental factors and benthic community dispersion, or that benthic dispersion is being driven by smaller scale biological driving forces, such as competition, predation and reproduction.

In conclusion, multivariate dispersion (a univariate metric) was able to capture and synthesize complex underlying multivariate gradients in coral reef benthic community characteristics across our study sites in American Samoa. In particular, the metric helped to highlight key differences in coral assemblages and their life-history strategies among dispersion categories. Similar community gradients for the other benthic groups (e.g. macroalgae) might be revealed by increasing their taxonomic resolution. The use of multivariate dispersion as a response metric could be further tested on temporal benthic community data to test whether it effectively captures shifts in successional states and community recovery following disturbance and the impacts of gradients in local human disturbance across broader spatial scales. Multivariate dispersion could be used as a synthetic data reduction method for monitoring coral reef benthic communities and has the potential to be used more broadly to understand community differences across other trophic levels that may exist within and between reefs.

## Data Availability

The dataset supporting this article can be found on GitHub at: https://github.com/alicelawrence2021/dispersion. Source code available at [96], and have been archived within the Zenodo repository: https://doi.org/zenodo.14725878. Supplementary material is available online [97].
